# Structural analysis and molecular substrate recognition properties of *Arabidopsis thaliana* ornithine transcarbamylase, the molecular target of phaseolotoxin produced by *Pseudomonas syringae*

**DOI:** 10.3389/fpls.2023.1297956

**Published:** 2023-12-18

**Authors:** Maciej Nielipinski, Agnieszka J. Pietrzyk-Brzezinska, Alexander Wlodawer, Bartosz Sekula

**Affiliations:** ^1^ Institute of Molecular and Industrial Biotechnology, Faculty of Biotechnology and Food Sciences, Lodz University of Technology, Lodz, Poland; ^2^ Center for Structural Biology, National Cancer Institute, Frederick, MD, United States

**Keywords:** urea cycle, arginine biosynthesis, halo blight disease, chlorosis, plant metabolism, antimetabolites, ornithine

## Abstract

Halo blight is a plant disease that leads to a significant decrease in the yield of common bean crops and kiwi fruits. The infection is caused by *Pseudomonas syringae* pathovars that produce phaseolotoxin, an antimetabolite which targets arginine metabolism, particularly by inhibition of ornithine transcarbamylase (OTC). OTC is responsible for production of citrulline from ornithine and carbamoyl phosphate. Here we present the first crystal structures of the plant OTC from *Arabidopsis thaliana* (*At*OTC). Structural analysis of *At*OTC complexed with ornithine and carbamoyl phosphate reveals that OTC undergoes a significant structural transition when ornithine enters the active site, from the opened to the closed state. In this study we discuss the mode of OTC inhibition by phaseolotoxin, which seems to be able to act only on the fully opened active site. Once the toxin is proteolytically cleaved, it mimics the reaction transition state analogue to fit inside the fully closed active site of OTC. Additionally, we indicate the differences around the gate loop region which rationally explain the resistance of some bacterial OTCs to phaseolotoxin.

## Introduction

**Figure 1:**
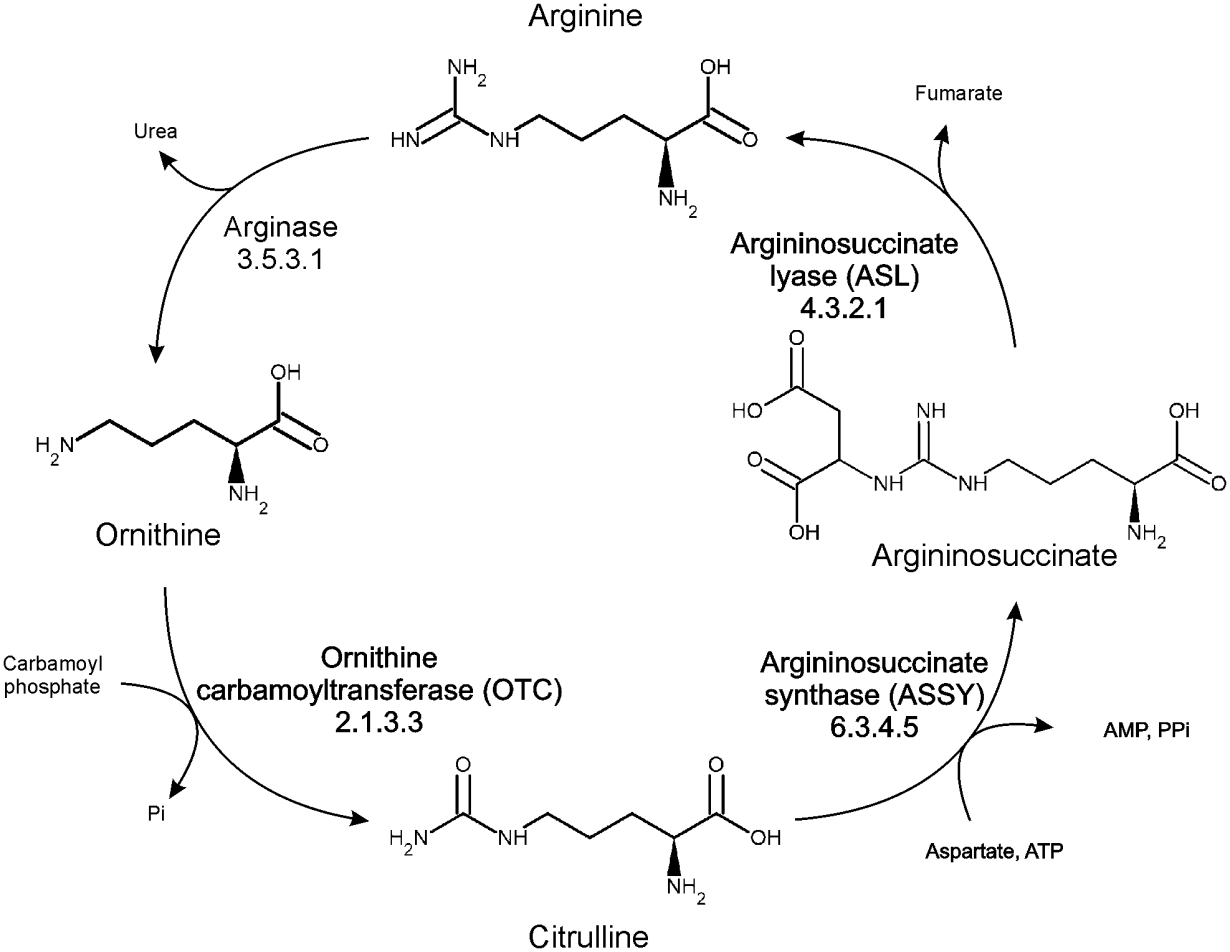
Organization of arginine metabolism in plants.

In higher plants, arginine metabolism plays a significant role (directly or via polyamines) in many physiological processes, including fruit ripening or stress response to abiotic and biotic factors (Kalamaki et al., 2009). Arginine biosynthesis in plants is carried out in plastids via the conversion of ornithine in the urea cycle (**Figure 1**). The first step of the cycle, catalyzed by ornithine transcarbamylase (ornithine carbamoyltransferase, OTC, EC 2.1.3.3), is the reaction of ornithine with carbamoyl phosphate to convert them into citrulline, with simultaneous phosphate release. The major difference in the utility of the urea cycle in plants in comparison to metazoans is that plants do not use it for the removal of nitrogen from amino acid catabolism. Instead, the urea cycle serves plants to recycle and distribute nitrogen and carbon (Winter et al., 2015). It is an important part of the metabolic response of diatoms to episodic nitrogen availability and may serve as a regulatory control point of its metabolism, since arginine is used as a major nitrogen storage form, and it is also a signal molecule (Esteban et al., 2016). Increased ammonia concentration boosts the transcript levels of urea cycle genes and concentration of intermediates of the cycle (Urra et al., 2022). In the catabolic part of the cycle, arginine is mobilized to feed polyamine, glutamate, and proline production (Kalamaki et al., 2009; Urra et al., 2022) – it can be degraded to ornithine and urea by hexameric, manganese-dependent arginase (Sekula, 2020), or decarboxylated to agmatine by arginine decarboxylase. Plants are the only known eukaryotes which produce polyamines via the agmatine route in a two-step conversion, utilizing a dimeric, propeller-like pentein - agmatine iminohydrolase (Sekula and Dauter, 2019) and helically shaped octameric *N*-carbamoylputrescine amidohydrolase (Sekula et al., 2016).

The arginine biosynthetic process is a target of the pathogenic strains of *Pseudomonas syringae*, which are responsible for various plant infections. Some *P. syringae* pathovars produce antimetabolite toxins which inhibit several enzymes of arginine and proline metabolism, including the biosynthetic part of the urea cycle (Arrebola et al., 2011). Several *P. syringae* toxins were recognized or suspected to cause or increase chlorosis or necrosis in infected plants (Arrebola et al., 2003). One of the best-characterized toxins is a sulfodiaminophosphinyl tripeptide, called phaseolotoxin. It consists of a homoarginine-alanine-ornithine tripeptide linked to a sulfodiaminophosphinyl moiety. Several *P. syringae* strains were found to produce phaseolotoxin, including pv. *phaseolicola*, pv. *actinidae*, and pv. *syringae* (Arrebola et al., 2003). Once the toxin invades a plant, it is converted to octicidine (PSORN), the major form found in infected plant tissues (Dayan and Duke, 2014), which is a competitive OTC inhibitor (Langley et al., 2000). Phaseolotoxin was also found to negatively affect the pyrimidine biosynthetic process (Steve and Zinmay Renee, 1981) due to citrulline deficiency (Durbin, 1991). The consequence of its action is reduction of ribosomal activity, a decrease in lipid synthesis, membrane permeability interference, and an accumulation of large starch grains in chloroplasts (Agrios, 2005). The widely known diseases caused by the infection by *Pseudomonas syringae* pathovars producing phaseolotoxin are halo blight in beans and canker in kiwi fruit. The main symptom recognized in plants infected by phaseolotoxin-producing strains is a chlorotic zone or a halo around the necrotic infection site (Arrebola et al., 2011). Infected plants exhibit pathological growth rates (Mitchell and Bieleski, 1977). The disease can reduce crop yield to nearly half in beans (Arnold et al., 2011).

OTC is an abundant protein, present in organisms across various kingdoms. Similarly to other enzymes forming the arginine-biosynthetic pathway (argininosuccinate synthase, ASSY, and argininosuccinate lyase, ASL), OTC also contains a chloroplast-targeting signal peptide (Urbano-Gámez et al., 2020). OTC is usually encoded by a single gene (Couchet et al., 2021). It belongs to the aspartate/ornithine carbamoyltransferase family of enzymes, which also includes aspartate transcarbamylase (ATC) and putrescine transcarbamylase (PTC). Transcarbamylase family regulates the urea cycle (arginine production) and *de novo* pyrimidine biosynthesis. Apart from the anabolic OTC that catalyzes ornithine-to-citrulline conversion, there are also catabolic orthologs, whose main function is the reversed reaction within the arginine deiminase pathway. The later orthologues function in lower organisms, where arginine is used to generate ATP (Shi et al., 2015). Interestingly, some of the *P. syringae* strains, such as *P. syringae* pv. *phaseolicola*, have two OTCs (*Ps*OTC and *Ps*ROTC, both anabolic) which exhibit different sensitivity to phaseolotoxin (Peet and Panopoulos, 1987). The gene of phaseolotoxin-insensitive OTC is most likely connected to a gene cluster involved in toxin production. The phaseolotoxin-insensitive OTC (*Ps*ROTC) provides an alternative source of arginine by acting as a functional replacement for housekeeping OTC, but also controls phaseolotoxin production through carbamylation of its precursor to nontoxic citrulline-alanine-homoarginine tripeptide (Chen et al., 2015).

Up to this date, no structure of plant OTC has been deposited in the Protein Data Bank (PDB), albeit numerous prokaryotic and eukaryotic OTC structures are known. Here we present structural analysis of *Arabidopsis thaliana* OTC (*At*OTC), characterized by X-ray crystallography. The analysis is based on two crystal structures of the ligand-bound *At*OTC complexed with ornithine (*At*OTC-ORN) and with carbamoyl phosphate (*At*OTC-CP). The structures provide the first experimental structural evidence of how the plant enzyme recognizes its substrates, giving insights into structural changes of the protein upon ligand binding. Additionally, we performed a thorough phylogenetic analysis of transcarbamylases, highlighting key features that determine sensitivity to phaseolotoxin, and thus the sensitivity to *P. syringae* infection.

## Results and discussion

### Phylogenetic analysis of aspartate/ornithine carbamoyltransferase superfamily

**Figure 2:**
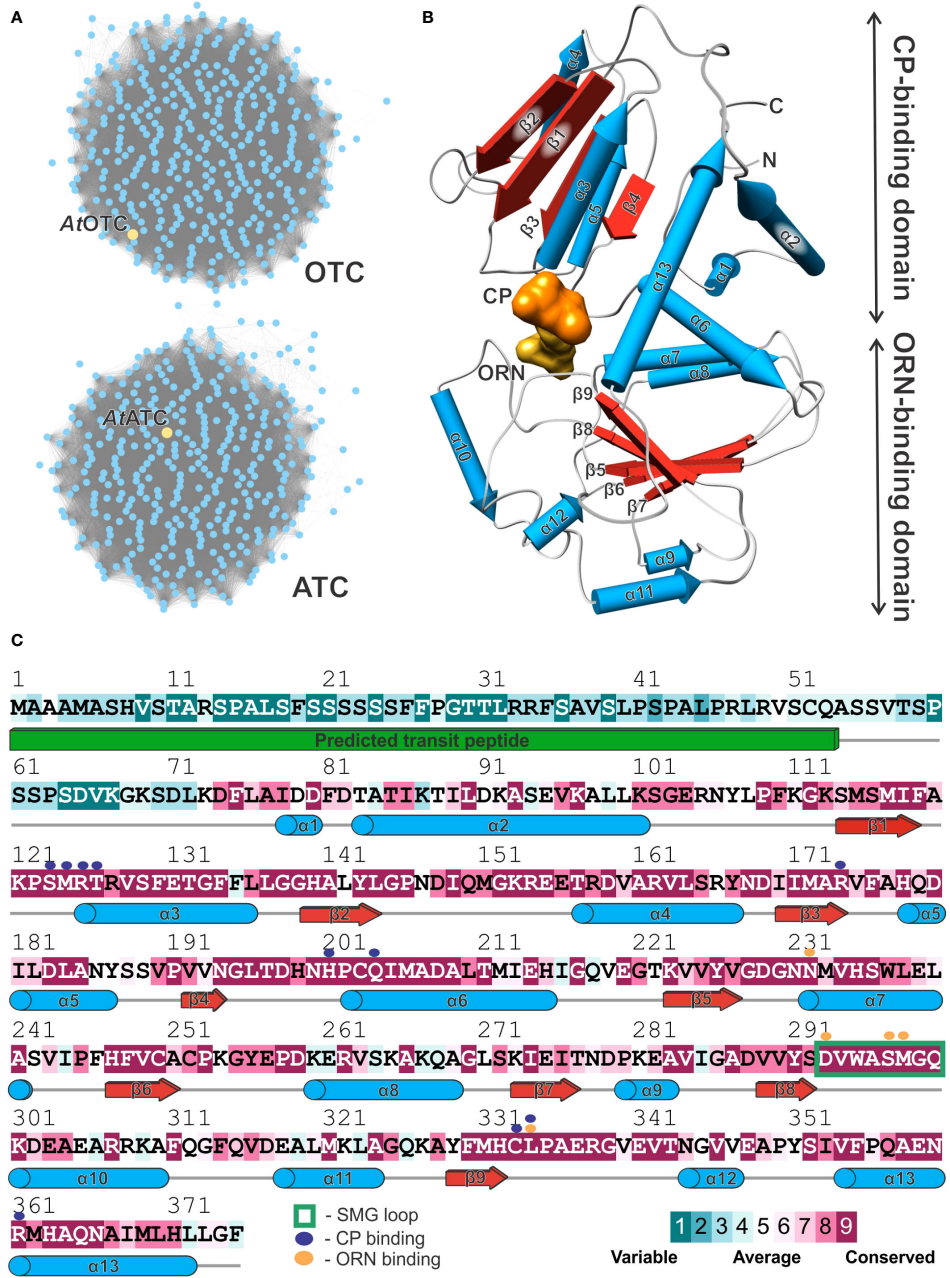
Plant ornithine transcarbamylases. **(A)** Sequence similarity network of *Viridiplantae* carbamoyltransferases (InterPro IPR036901 family). Yellow nodes depict *At*OTC (UniProt ID: O50039) and *At*ATC (UniProt ID: P49077) sequences in the group of OTCs and ATCs, respectively. **(B)** Structure of the *At*OTC subunit presenting secondary structure elements (pipes-and-planks model) and division into CP-binding and ORN-binding domains; binding sites of CP and ORN are presented as solid surface (orange and yellow, respectively). **(C)** Sequence conservation of *Viridiplantae* OTCs mapped on the *At*OTC sequence (UniProt ID: O50039); In total, 389 were aligned and analyzed. Secondary structure elements recognized in the *At*OTC structure are shown as blue pipes (helices); red arrows (β-strands), and grey lines (loop regions); the green solid cuboid depicts the predicted transit peptide which was truncated at position 53. Residues interacting with CP and ORN in the active site are marked with purple and orange dots (see legend), flexible SMG loop is marked with a green rectangle.

Flagship examples of proteins in the aspartate/ornithine carbamoyltransferase superfamily are OTCs and ATCs. We analyzed sequences of *Viridiplantae* clade of the superfamily (IPR036901) from the InterPro database (Finn et al., 2017). The sequence similarity network shows that OTC and ATC are the only representatives of the family in plants (**Figure 2A**). They are equally represented (389 vs. 377 of OTC and ATC sequences, respectively). The whole family follows the same general fold with two domains, a CP-binding and amino-acid-binding domain, similar to that observed in the determined structure of *At*OTC (**Figure 2B**).

Looking at the sequence conservation of plant OTCs (**Figure 2C**), based on the ConSurf analysis (Yariv et al., 2023), the conserved CP-binding domain starts shortly before helix α1, around residue D74 of *At*OTC. It corresponds with an observation in our crystal structure – residues before L72 are disordered, although the *At*OTC construct used for the study started at Q53 (see Materials and Methods). There are four highly conserved motifs throughout OTCs engaged in substrate binding: (1) β1-α3 loop containing _123_SMRTR_127_ motif (numbers refer to sequence position in *At*OTC), (2) region around α6 containing _201_HPCQ_204_ motif, both participating in CP binding, (3) the loop preceding α10 (starting at position D293) with _297_SMG_299_ motif, participating in ornithine binding, and (4) the loop following β9 with _332_HCLP_335_, engaged in the binding of both substrates (**Figure 2C**). In ATC, these motifs are STRTR, HPTQ, YQTR, and HPLP (**Supplementary Figure S1**). Naturally, the major difference is the region responsible for interaction with ORN/ASP substrate: SMG (OTC) vs. YQTR (ATC). Lower sequence conservation of plant OTCs can be observed in the regions of α2, the terminal part of α6 with the following loop, α8, α10, α11, and α12. These regions involve surface residues which do not participate in interdomain or substrate interactions.

**Figure 3:**
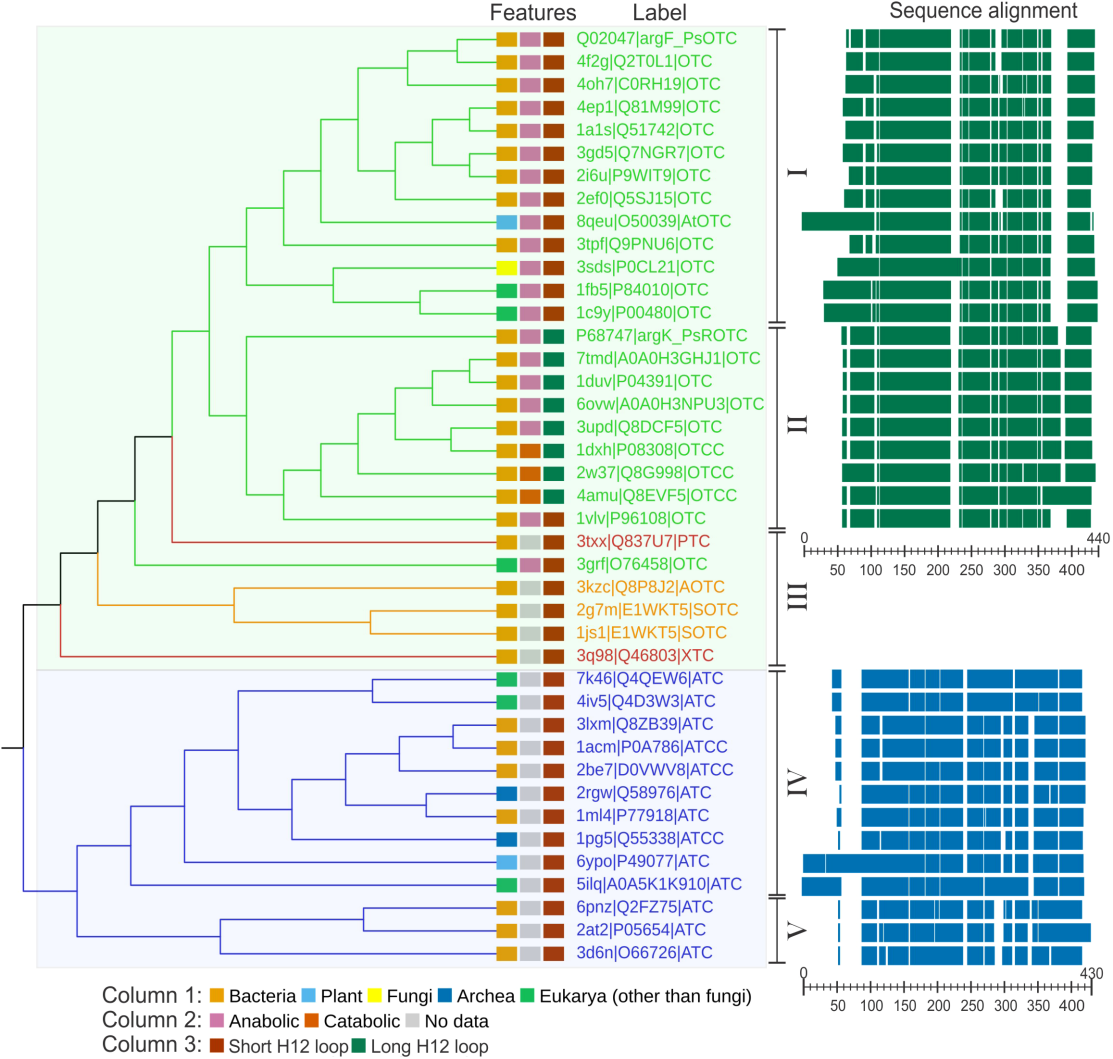
Phylogenetic analysis of carbamoyltransferases. Cladogram of carbamoyltransferases structures along with sequence alignment of OTCs and ATCs. Labels are given in the following manner: PDB ID| UniProt IDProtein designation. Protein designations are as follows: OTC, ornithine carbamoyl transferase; ROTC, phaseolotoxin resistant OTC; OTCC, catabolic OTC; PTC, putrescine carbamoyltransferase; AOTC, acetylornithine carbamoyltransferase; SOTC, succinylornithine carbamoyltransferase; XTC, putative carbamoyltransferase; ATC, aspartate carbamoyltransferase; ATCC, catalytic unit of ATC Branches and labels are colored by protein type: OTC (green), ATC (blue), modified ornithine carbamoyltransferases (orange), other carbamoyltransferases (red). Features of each protein (kingdom; anabolic/catabolic type; α12 type) are marked in color-coded rectangles (see legend below the cladogram). The Roman numbers on the right side of the cladogram represent the clades described in the text. The right panel shows a schematic representation of the sequence alignment; scales indicate the position of the alignment. The references of the entries used in the analysis: 1A1S (Villeret et al., 1998), 1ACM (Stebbins et al., 1992), 1C9Y (Shi et al., 2000), 1DUV (Langley et al., 2000), 1DXH, 1FB5 (De Gregorio et al., 2003), 1JS1 (Shi et al., 2002), 1ML4 (Van Boxstael et al., 2003), 1PG5 (De Vos et al., 2004), 1VLV, 2AT2 (Stevens et al., 1991), 2BE7 (De Vos et al., 2007), 2EF0, 2G7M (Shi et al., 2007), 2I6U (Sankaranarayanan et al., 2008), 2RGW (Vitali et al., 2008), 2W37 (de las Rivas et al., 2009), 3D6N (Zhang et al., 2009), 3GRF (Galkin et al., 2009), 3KZC (Shi et al., 2005), 3Q98 (Li et al., 2011), 3SDS (Hewitt et al., 2011), 3TPF (Shabalin et al., 2012), 4AMU (Gallego et al., 2012), 4F2G (Baugh et al., 2013), 5ILQ (Lunev et al., 2016), 6YPO (Bellin et al., 2021), 8QEU (this work).

Analyzing sequences of transcarbamylases for which crystal structures are available (**Figure 3**) it can be seen that OTCs (clade highlighted in green on the tree in **Figure 3**) and ATCs (clade highlighted in blue on the tree in **Figure 3**) form clearly distinguishable subfamilies, not only in plants but also in other kingdoms. Eukaryotic ATCs and OTCs have extended N termini, due to the presence of a signal peptide responsible for the translocation of the protein into mitochondria or chloroplasts. In general, the ATC group can be divided into two distinguishable groups. The main difference between them is the length of a helix around positions 290-300 in the alignment. Group V, with the shorter helix, comprises 3 bacterial structures, and group IV, with the longer helix, includes 10 structures belonging to various domains of life. When it comes to the group of OTCs, it is more diverse – there are two main groups (group I and II) and six, clearly divergent enzymes (group III). Five of the proteins belonging to group III are prokaryotic proteins that recognize substrates different than ornithine, namely: putrescine (PDB ID: 3TXX), acetylornithine (PDB ID: 3KZC; Shi et al., 2005), succinylornithine (PDB ID: 2G7M, Shi et al., 2007; 1JS1, Shi et al., 2002), and an unknown substrate (PDB ID: 3Q98; Li et al., 2011). The sixth protein is OTC from protist *Giardia intestinalis* (PDB ID: 3GRF; Galkin et al., 2009), which targets ornithine, despite remaining outside of the main two OTC clades. Representatives of all domains of life can be found in the anabolic OTC group (group I), including *At*OTC. The sequence of *Ps*OTC (sensitive to phaseolotoxin) is assigned to this group as well. Group II of OTCs is formed exclusively by bacterial proteins, all of which share a common trait of having an extended region corresponding to the post-α12 loop, or even having an additional helix in place of this loop (around position 360-380 of the alignment, **Figure 3**). It contains both, anabolic and catabolic, OTC types. Interestingly, *Ps*ROTC (phaseolotoxin-insensitive type) is assigned to this group as well. It exhibits traits similar to catabolic enzymes with an extended α12 loop, which may decide on the insensitivity to phaseolotoxin.

### *At*OTC structure

**Figure 4:**
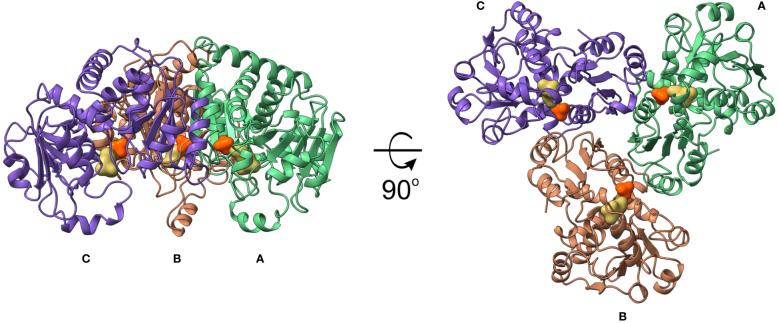
*At*OTC crystal structure. Trimeric assembly of *At*OTC in cartoon representation; CP and ORN binding sites are shown as orange and yellow solid surfaces, respectively.

*At*OTC, like many other OTCs, forms a trimer, with subunits arranged around a non-crystallographic three-fold axis (**Figure 4**). Each subunit has a surface area of ~13000 Å^2^ and the interface between subunits is ~1200 Å^2^, formed by approximately 30 residues from the subunit neighbor. Interfaces between protomers are formed by the β1 (together with a few preceding residues), long fragment involving β2 and α4 (residues 138-169) from one subunit, which interact with the loop between α2-β1, α3 helix, β2 together with the following loop, and the fragment of α12-α13. The residues in a β1-α3 region, on top of the interface, take part in CP binding, as well (**Figure 2C**). There are 36-38 hydrogen bonds and salt bridges formed on the interfaces between subunits.

The *At*OTC subunit, similarly to other OTCs, is organized in two α/β domains (**Figure 2B**): N-terminal (CP-binding) and C-terminal (ORN-binding) domain. Each has a Rossman-like fold with a parallel β-sheet surrounded by helices. CP-binding domain has a total of 7 helices, which flank the 4-stranded β-sheet (two helices from the outer side, and a 5-helical bundle from the interdomain side). The bundle is formed by α1-α3, α6, and the C-terminal helix α13, which crosses the domain interface, reaching the N-terminus. The ORN-binding domain is smaller; it is built of a total of 6 helices and a 5-stranded β-sheet.

*At*OTC has a similar quaternary structure to the other plant representative of carbamoyltransferases, *At*ATC (PDB ID: 6YVB; Bellin et al., 2021). RMSD of the superposed structures with bound CP is 2.3 Å with 822 of 916 residues aligned. The binding site of CP in *At*OTC has nearly identical architecture to that of *At*ATC, with two highly conserved motifs present – S*RTR and H*LP (* denotes differences between the two enzymes). The differences are the change of R154 and C333 of the *At*OTC to S163 and P349 in *At*ATC. Also, R361 in *At*OTC corresponds to G375 in *At*ATC, thus disabling interaction with CP in the later enzyme. However, the position of bound CP in both proteins is virtually the same. In both proteins loop β2-α4 from the neighboring subunit is engaged in CP stabilization.

### Active site

**Figure 5:**
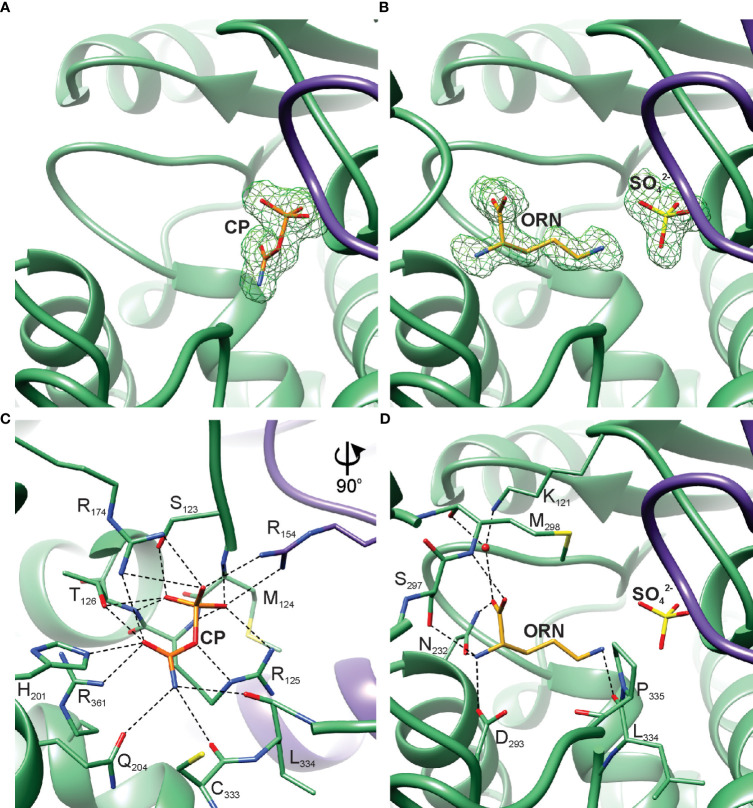
*At*OTC active site. Omit map contoured at 3 σ around the bound ligands: **(A)** CP in *At*OTC-CP structure, **(B)** ORN and SO_4_^2-^ in *At*OTC-ORN structure. Detailed interactions of the substrates bound in the active site of *At*OTC: **(C)** CP and **(D)** ORN. Chains A and B of the structures are shown in green and violet, respectively.

The interactions of *At*OTC with substrates were investigated based on two experimental crystal structures of the *At*OTC, complexed with ORN or CP (*At*OTC-ORN and *At*OTC-CP). The complexes were obtained by cocrystallization. The resolution of both structures is near 1.5 Å and the quality of electron density maps for bound ligands (**Figures 5A, B**) allowed us to determine the precise position of the ligands bound in the active site; the ligands were refined with full occupancy. In the *At*OTC-ORN complex, we identified additional SO_4_^2-^ ion (lithium sulfate was one of the crystallization buffer components) bound in the CP-binding location (**Figure 5B**). The sulfate ion has an almost identical conformation as the phosphate moiety of CP in *At*OTC-CP structure.

The active site of *At*OTC is located inside a cleft formed at the domain interface (**Figure 2B**). The residues participating in substrate binding (**Figures 5C, D**) belong to highly conserved OTC motifs (**Figure 2C**). CP-binding cavity is deeper, and it is created with the contribution of the residues from a loop between β2 and α4 of the neighboring subunit (**Figure 5C**). Three arginine side chains located deep inside the pocket create a patch of positive potential, which attracts the phosphate group of CP or the sulfate from the crystallization buffer. The phosphate moiety of the bound CP in the *At*OTC-CP structure creates an extensive network of hydrogen bonds with surrounding residues (**Figure 5C**), including interactions with the _123_SMRT_126_ motif of the β1-α3 region and R154 of the β2-α4 loop from the other subunit. The carbamoyl moiety of CP is stabilized by H201 and Q204 of helix α6, R361 of helix α13 and the main chain of the loop β9-α12 (carbonyl oxygens of C333 and L334), as well as a hydroxyl group of T126 of the _123_SMRT_126_ motif. R174 from β3 interacts with both the carbamoyl and phosphate groups via its side chain. ORN binding site in the C-terminal domain exhibits significant structural adaptability. Its boundaries are shaped by a highly mobile region around helix α10 which, together with the preceding loop, regulates the size of the cavity and the substrate accessibility (see below). It contains a highly conserved _297_SMG_299_ motif. The α-amine group of ORN creates three hydrogen bonds with side chains of N232, D293, and S297 (**Figure 5D**). The carboxylic group directly interacts with the N232 side chain and M298 backbone. It also creates water-mediated H-bonds with the carbonyl oxygen of M298 and the side-chain amine of K121 (**Figure 5D**). The ϵ-amine of ORN is hydrogen-bonded with the carbonyl oxygen of L334.

Superposition of both determined structures shows that the Nϵ of ORN is placed ideally above the carbamoyl moiety of CP (the measured Nϵ_(ORN)_-C_(CP)_ distance is 2.4 Å), supporting the generally accepted reaction mechanism (Couchet et al., 2021). The reaction proceeds via an ordered bi-bi mechanism and follows Michaelis–Menten kinetics. At first, *At*OTC needs to bind CP to form a binary complex, compensating positive potential focused around the CP-binding cavity. As suggested by (Goldsmith et al., 1991), ORN is most likely bound as a zwitterion. It is “locked” by the _297_SMG_299_ motif, which stabilizes the transition state and secures the position of the ϵ-amine group close to CP. The reaction is initiated by a nucleophilic attack on the central carbon of CP, forming a tetrahedral intermediate. One of the phosphate oxygen atoms of CP is likely to be involved in accepting a proton from Nϵ atom of ORN (Sankaranarayanan et al., 2008). Ultimately, the intermediate breaks down into citrulline and a phosphate ion. Products can be released by the movements of the SMG loop, with the initial citrulline departure followed by phosphate release.

### Conformational changes of the SMG loop and helix α10

**Figure 6:**
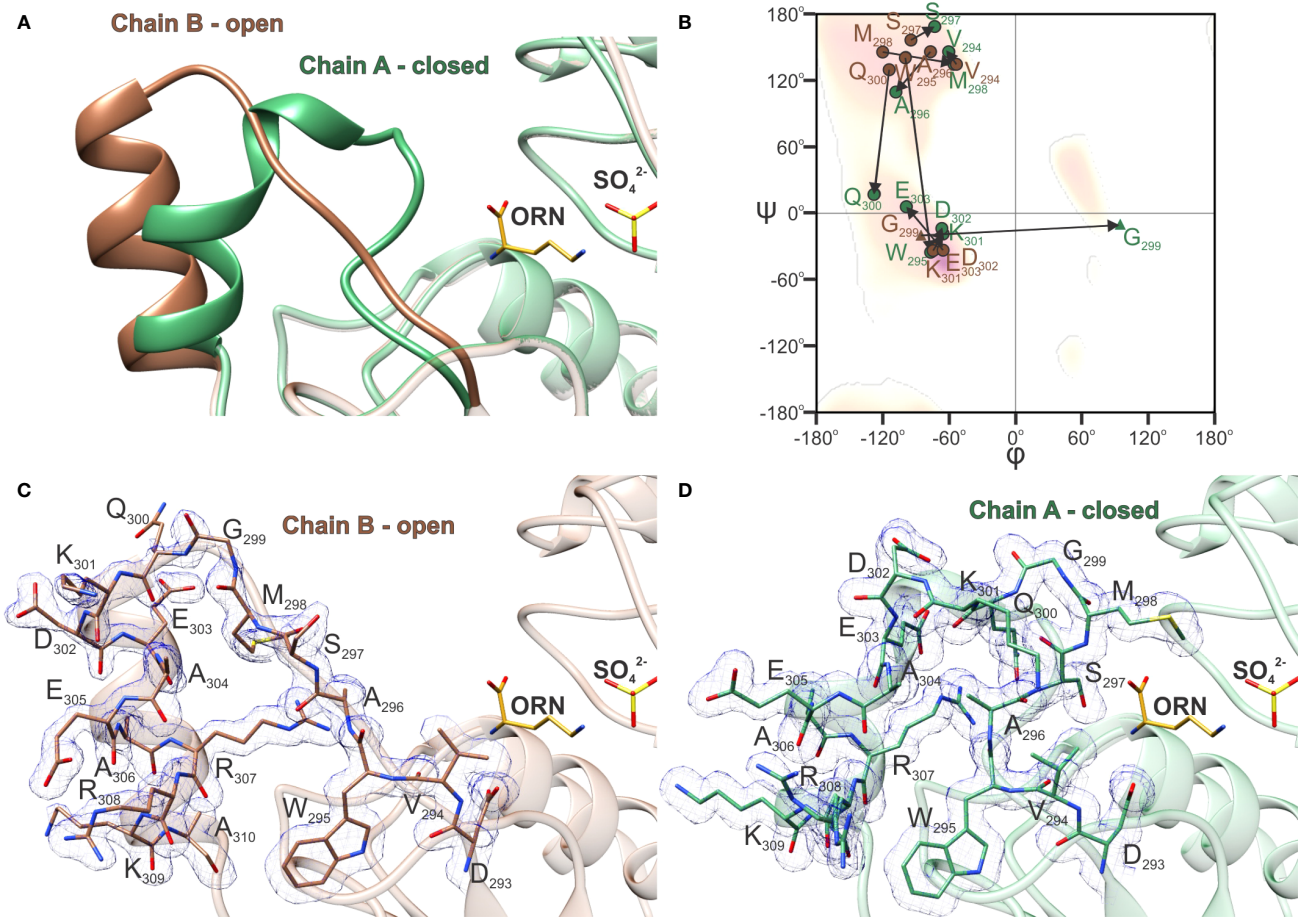
Open-to-closed transition of *At*OTC. **(A)** Comparison of SMG loop and helix α10 conformation in chain A (green) and chain B (brown) of *At*OTC-ORN structure (PDB ID: 8QEU) representing closed and open conformations of the SMG loop. **(B)** Ramachandran difference plot (Kleywegt plot) of the SMG loop of chain A (green) and chain B (brown) of *At*OTC-ORN structure (residues 294-303); residues are marked with circles (any residue) and triangles (glycine); arrows depict the change of φ and ψ angles during the transition from open to close conformation. Electron density maps 2*F_o_-F_c_* displayed at 1 σ for the SMG loop and helix α10: **(C)** in chain A and **(D)** chain B of *At*OTC-ORN structure.

Investigation of the crystal structures presented here showed that the region around α10 exhibits high conformational mobility. In the *At*OTC-CP structure, where the ORN binding cleft remains empty, we were not able to trace the conformation of the SMG loop, which naturally participates in ORN binding. This region was fully structured only when ORN occupied the active site. A thorough analysis of the *At*OTC-ORN structure showed that two alternative conformations of the α10 region were captured in chains A and B (**Figure 6A**). The conformational changes include the SMG loop and the helix α10, namely residues 294-304 (**Figure 6B**). In a relaxed (open) state the loop fragment is straight and sharply turns around G299 to form a 3-turn helix α10, extending from residue K301 to A310 (**Figures 6A, C**). Torsion angles of the loop residues (except for G299) point to the β-sheet conformation on the Ramachandran plot (**Figure 6B**). The transition from open to closed conformation is accompanied by significant backbone rearrangements (**Figures 6B, D**). The loop bends, residues 301-303 move out from the helix α10 to form one turn of the 3_10_ helix, and helix α10 significantly shifts its position (**Figure 6A**). The difference in the conformation is observed starting from V294 and W295, backbones of which are shifted by ~2 Å. The V294 side chain is rotated by ~90°, but there is a minor change of the torsion angles of its backbone. On the other hand, the carbonyl oxygen of W295 is flipped and interacts via H-bond with the R307 side chain when the enzyme is in a closed conformation. This movement of W295 is accompanied by rotation of A296. The carbonyl oxygen of A296 forms an H-bond with the K301 side chain. Bending a loop around W295-A296 gives a possibility of S297 (of the _297_SMG_299_ motif) to be placed 7 Å away from the open position to easily interact with the bound ORN inside the active site cavity. At the same time, the carbonyl oxygen of S297 creates a hydrogen bond with the backbone amine of Q300. Also, the flipped side chain of Q300 is H-bonded with backbone N of S297 and additionally interacts with R307. During the open-to-closed transition, M298 is placed above the active site ~10 Å away from its initial place. Also, a part of helix α10 unwinds to break a hydrogen bond network in a helical fragment. In the closed conformation, residues 302-304 are off the α10 helix axis by ~25°. Additionally, characteristics for α-helical O-N hydrogen bonds are preserved only for the A304-A310 fragment, resulting in the helix α10 being shortened by one turn. The backbone of the fragment of K301-E303 is bent and corresponds to the characteristics of 3_10_ helix. The closed conformation of the SMG loop is stabilized by newly created H-bonds of bent loop residues (W295, A296, S297, Q300, K301, and water-mediated bonds of M298, V294).

The high structural mobility of the SMG loop is also characteristic for other OTCs. A comparison with the human enzyme (*Hs*OTC) bound to bisubstrate analog PALO (PDB ID: 1OTH; Shi et al., 1998), shows that the conformation of the closed SMG loop of *Hs*OTC is highly similar to that observed in *At*OTC, despite significant sequence differences: _294_VWASMGQKDEAE_305_ in *At*OTC and _264_TWISMGREEEKK_275_ in *Hs*OTC. Additionally, the SMG loop of *Hs*OTC in its unbound (PDB ID: 1FVO; Shi et al., 2001) or bound only to CP (PDB ID: 1EP9; Shi et al., 2001) forms, is in the open conformation, somewhat similar to the conformation of the B-chain of *At*OTC structure.

### Structural basis of OTC inhibition by phaseolotoxin

OTC activity is inhibited competitively by phaseolotoxin produced by *Pseudomonas syringae* pv. *phaseolicola*, especially by the product of its hydrolysis, octicidine (PSORN) - N delta-(N’-sulfodiaminophosphinyl)-L-ornithine. PSORN exhibits a high structural resemblance to the intermediate of OTC transcarbamoylation reaction (Langley et al., 2000), with the diaminophosphinyl group presenting tetrahedral geometry.

**Figure 7:**
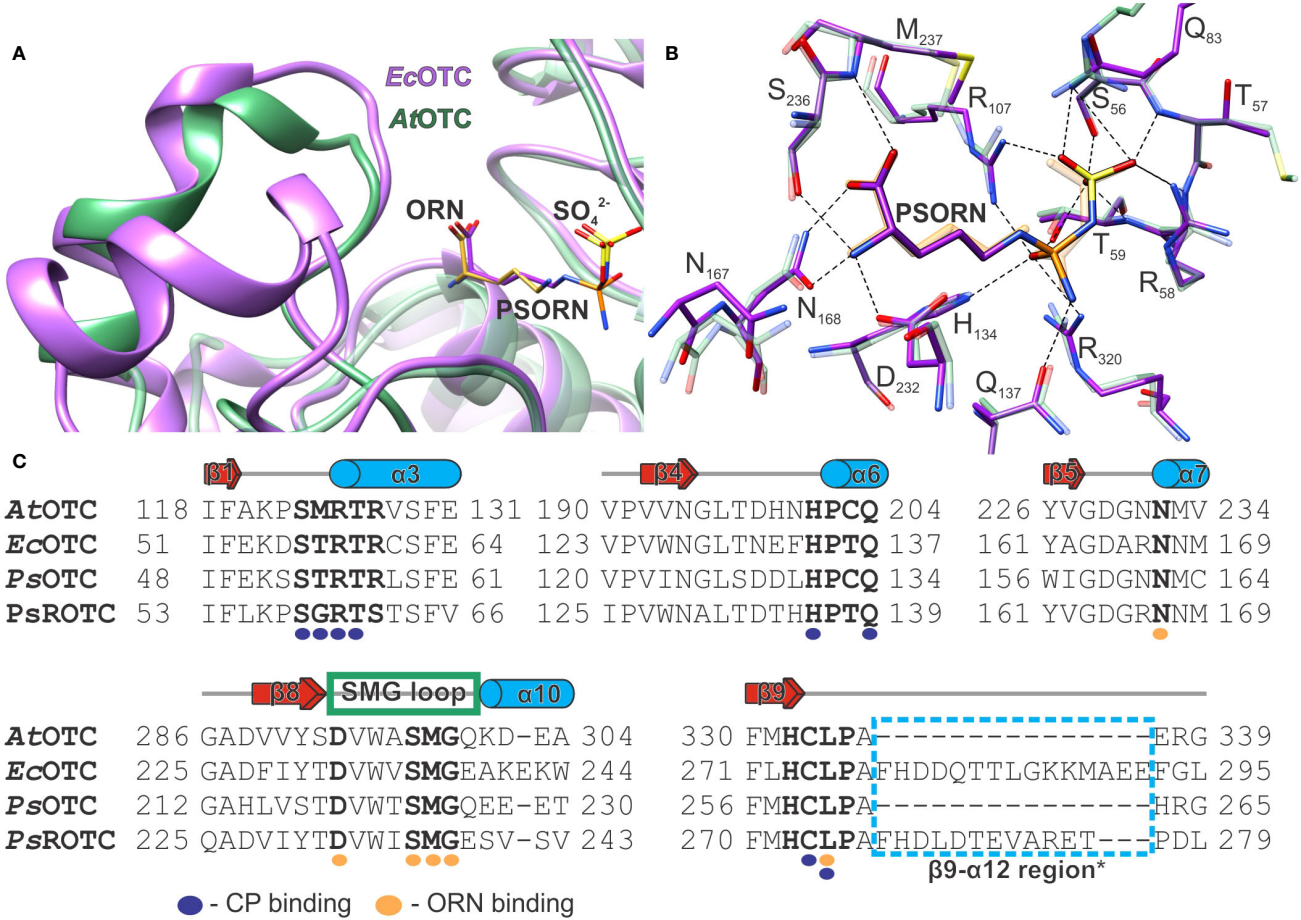
The analysis of OTC inhibition by PSORN. **(A)** Superposition of *At*OTC-ORN structure (PDB ID: 8QEU; green ribbon) with *Ec*OTC-PSORN (PDB ID: 1DUV; violet ribbon); ORN is shown as orange sticks; PSORN is violet. **(B)** the interactions of PSORN (dashed lines) in the active site of *Ec*OTC (PDB ID: 1DUV; violet sticks); the corresponding residues of the superposed *At*OTC structure are shown as semi-transparent green sticks; ORN and CP binding location are depicted in orange; PSORN is violet. **(C)** Sequence alignment of *At*OTC (UniProt ID: O50039), *Ec*OTC (UniProt ID: P04391); *Ps*OTC (UniProt ID:Q02047), *Ps*ROTC (UniProt ID: P68747) only the neighboring regions of the conserved motifs and ORN binding (bold residues) are shown; CP and ORN binding residues are marked with purple and orange circles below the alignment; secondary structure elements refer to *At*OTC structure (see **Figure 1** caption); an additional structural element of *Ec*OTC and *Ps*ROTC (β9-α12 region*) is marked with a blue dashed line box.

The structure of the *E. coli* OTC complex with PSORN (PDB ID: 1DUV) is available (Langley et al., 2000). The toxin binds to the enzyme competitively to ORN and CP in a non-covalent manner. Superposition with our *At*OTC-ORN complex shows that ORN and sulfate align well with PSORN (**Figure 7A**). Simultaneously, CP in the *At*OTC-CP complex superposes with sulfodiaminophosphinyl moiety of PSORN, with the difference that the carbamoyl moiety of CP is planar. The hydrogen-bonding network of PSORN is almost identical to that of ORN and CP bound in *At*OTC (**Figure 7B**). Sulfodiaminophosphinyl moiety of PSORN interacts with residues of two neighboring subunits in the CP-binding cleft, while the amine and carboxyl group interact with SMG loop, which is in a closed conformation. This strongly suggests that PSORN inhibition of *At*OTC should be the same as it is in *Ec*OTC. Looking at the structures the explanation of why PSORN is a more potent inhibitor than the full phaseolotoxin is rather obvious. PSORN, which lacks alanine-homoarginine dipeptide, fits the active site, mimicking the physiological intermediate state of the OTC reaction. The enzyme is “locked” in a closed conformation of the SMG loop-α10 region. OTC is not able to degrade diaminophosphinyl group of PSORN. Significantly larger phaseolotoxin, with additional alanine-homoarginine dipeptide attached to the carboxyl group of PSORN could penetrate the active site, but only when the SMG loop-α10 is in an open conformation. Otherwise, the toxin would create serious steric hindrance with the _297_SMG_299_ region. Binding of the toxin to the exposed active site should be in fact less stable than the binding of PSORN within the “locked” pocket.

The fact that the substrate-binding region of various OTCs is highly conserved raises a question why some organisms are less sensitive to the toxin. One explanation would be that some organisms may not be able to transport the toxin inside their cells via oligopeptide permease (Staskawicz and Panopoulos, 1980), or may not have peptidases that degrade the phaseolotoxin to PSORN. However, some *Pseudomonas* strains, such as *P. syringae pv. phaseolicola*, produce two OTCs (*Ps*OTC and *PsR*OTC) (Jahn et al., 1985), which exhibit significantly different sensitivity to phaseolotoxin (Peet and Panopoulos, 1987). Therefore, OTC sensitivity to the toxin may also be related to some structural features of the enzyme.

The alignment of *At*OTC, *Ps*OTC (argF), and *Ps*ROTC (argK) shows that *At*OTC and *Ps*OTC share 41% identity and both share ~35% identity with *Ps*ROTC. All three enzymes have the structural motifs HCLP and SMG, as well as other amino acids involved in the physiological substrate binding (**Figure 7C**). A difference can be observed in the CP-binding site, in the S*RTR motif (SMRTR in *At*OTC and STRTR in *Ps*OTC), where two residues are changed to G and S in *Ps*ROTC, forming SGRTS. While the CP-binding domains of both *Ps*OTC and *Ps*ROTC have different amino acid composition than *At*OTC, their size deduced from sequence alignment is comparable, as no gaps are present. On the other hand, in the ornithine-binding domain, the region corresponding to the β9-α12 region, following the HCLP motif, has 12 additional residues in *Ps*ROTC in comparison to *At*OTC and *Ps*OTC. Structures resembling that fragment can be found in some other OTCs, like *Ec*OTC, where they form an additional helix (**Figure 7A**). Interestingly, in *Ec*OTC this fragment is even longer than it is in *Ps*ROTC by three additional residues. Looking at the available structural data, the mentioned additional fragment of *Ps*ROTC should be placed near the SMG loop, like it is in *Ec*OTC structure. Moreover, the region following the SMG motif is different in the discussed proteins (**Figure 7C**). In *At*OTC it is QKDEA, which is similar to QEEETA in *Ps*OTC. However, in *Ps*ROTC the sequence of this fragment is significantly changed to ESVSV. In *Ec*OTC (also with additional helix), the fragment has the sequence EAKEKW. That additional structural element following the HCLP motif, together with differences in the SMG loop region, may decide on a different opening mechanism of the SMG loop of *Ps*ROTC. This would give a possible explanation of the insensitivity of *Ps*ROTC to phaseolotoxin. An incomplete opening of the SMG loop would result in steric hindrance for the toxin to infiltrate the active site. Smaller substrates, CP and ORN, would fit the active site anyway, with only minor difficulties, explaining a lower affinity of *Ps*ROTC towards CP than the affinity presented by *Ps*OTC (Jahn et al., 1987). This would also explain why PSORN is a much more potent inhibitor than phaseolotoxin, since the latter can act only on the fully open enzyme. Therefore, the high potency of PSORN can be attributed to a good fit to the CP and ORN binding cavities which are locked by the SMG region.

## Conclusions

This work presents the first crystal structures of a plant OTC. As the structures represent the complexes of *At*OTC and ORN or CP, it was possible to describe and analyze in detail the interactions between the enzyme and its substrates. Additionally, two states of the SMG loop were captured (opened/closed). The concerted conformational changes of the SMG loop and helix α10, were observed when ORN entered the active site. Furthermore, the phylogenetic analysis and structural comparisons of *At*OTC and other aspartate/ornithine carbamoyltransferases were performed and the conserved motifs were identified in plant OTCs. Finally, we indicated the structural differences between OTCs inhibited by phaseolotoxin or PSORN and those resistant to the toxin. One of the utilities that arise from this study is a possibility of developing plant strains that produce phaseolotoxin-resistant OTC. The differences in the SMG loop region plus additional structural elements in its close vicinity may decide on the insensibility to the toxin of some bacterial OTCs.

## Materials and methods

### Cloning, overexpression, and purification of *At*OTC

*Arabidopsis thaliana* complementary DNA was obtained by isolation of RNA from plant leaves with RNeasy Plant Mini Kit (Qiagen) and the use of SuperScirpt II reverse transcriptase (Life Technologies) in connection with oligo dT primers. Polymerase chain reaction was used to isolate the open reading frame of *At*OTC (Ordered Locus Name: AT1G75330; UniProt ID: O50039). Primers for PCR reaction were designed to obtain the *At*OTC sequence starting from codon 53. Used primers were as follows:

TACTTCCAATCCAATGCCTCCTCCGTCACTTCGCCTTCTT (forward)
TTATCCACTTCCAATGTTAAAAGCCGAGCAAGTGAAGCATTATAGCA (reverse)

To clone the obtained cDNA into the pMCSG68 vector (Midwest Center for Structural Genomics) ligase-independent cloning protocol was used (Kim et al., 2011). Transformation of *Escherichia coli* BL21 Gold competent cells (Agilent Technologies) was done with approximately 60 ng/µL of vector. The concentration of the vector was checked by spectrophotometric absorbance measurement at 260 and 280 nm. To check the correctness of the obtained cDNA, DNA sequencing has been performed.

Inoculum for protein overexpression was prepared by overnight culture of transformed *E. coli* in 15 mL LB medium with 150 µg/ml of ampicillin. Protein production was conducted using 1 L of freshly prepared auto induction medium - LB broth base (FORMEDIUM Ltd.) with 150 µg/ml of ampicillin. After inoculation bacteria were cultured for 4 h at 37°C and shaken at 180 RPM. After that time culture was cooled to 16°C and left for 16 h. Afterwards culture was pelleted by centrifugation (2800 g, 20 min) and pellets were resuspended in binding buffer (50 mM HEPES pH 7.8, 500 mM NaCl, 1 mM TCEP, 20 mM imidazole). Suspended cells were placed in ice/water bath and disintegrated by sonification (60 cycles, 4 second bursts with 26 seconds interval). Obtained cell debris was pelleted by centrifugation (19000 g, 20 min). Supernatant was then transferred to a column packed with 5 ml of HisTrap HP resin (GE Healthcare) coupled with Vac-Man (Promega). The resin was washed five times with the binding buffer. Finally, the protein was eluted with 20 mL of the elution buffer (50 mM HEPES pH 7.8, 500 mM NaCl, 1 mM TCEP, 400 mM imidazole).

Obtained protein solution was then dialyzed overnight against dialysis buffer (50 mM HEPES, 500 mM NaCl, 1 mM TCEP) at 4°C. In parallel to dialysis cleavage of His_6_-tag was conducted by the addition of His_6_-tagged Tobacco Etch Virus (TEV) protease (final concentration 0.1 mg/ml). Separation of His_6_-tag and TEV protease was conducted on HisTrap HP resin by eluting *At*OTC with 10 ml of binding buffer. The obtained protein solution was concentrated using Vivaspin^®^ 20 concentrators (Sartorius) to the volume of 2 mL. The final step of purification was carried out by size-exclusion chromatography on HiLoad Superdex 200 16/60 (GE Healthcare) coupled with ÄKTA pure FPLC system (GE Healthcare) using the following buffer: 50 mM HEPES pH 7.8, 100 mM NaCl, 50 mM KCl, 1 mM TCEP. Collected purified fractions were concentrated to approximately 20 mg/mL using the same concentrators mentioned before. Concentration was determined by spectrophotometric absorbance measurement at 280 nm with MW 35500 Da and extinction coefficient 24410.

### Crystallization and data collection

Initial screening was carried out using Index Screen (Hampton) and the sitting drop method. The best crystals in screen were grown in Bis-Tris pH 6.5, 0.2 M Li_2_SO_4_ and 25% PEG 3350. Complexes with ornithine and carbamoyl phosphate were obtained by co-crystallization with 20 mM of respective substrates. After optimization *At*OTC with ornithine was grown in HEPES 0.1 M pH 6.0, 0.3 M Li_2_SO_4_, 16% PEG 3350 and *At*OTC with CP was grown in HEPES 0.1 M pH 6.0, 0.3 M Li_2_SO_4_ and 26% PEG 3350, both with hanging drop method. Crystals were grown at approximately 20°C and formed after one week. Both types of crystals were cryoprotected with PEG 400 before flash-freezing in liquid nitrogen.

Diffraction data were collected at BL 14.1 beamline at BESSY II Light Source in Helmholtz-Zentrum Berlin (Mueller et al., 2015). Diffraction data were processed using XDS (Kabsch, 2010). Data processing statistics are given in **Table 1**.

**Table 1:** Data collection and refinement statistics.

Structure:	*At*OTC-ORN	*At*OTC-CP
Data collection		
Beamline	BL 14.1 BESSY, Berlin	BL 14.1 BESSY, Berlin
Wavelength (Å)	0.9184	0.9184
Temperature (K)	100	100
Space group	*C* 2 2 2_1_	*C* 2 2 2_1_
Unit cell parameters *a, b, c* (Å)	89.3 155.4 189.5	89.2 154.6 191.8
Oscillation range (°)	0.3	0.3
Resolution (Å)	49.02-1.50 (1.59-1.50)	44.67- 1.55 (1.64-1.55)
Reflections collected/unique	1529963/208445	1074205/194853
Completeness (%)	99.6 (98.9)	99.2 (97.5)
Multiplicity	7.33 (7.14)	5.51 (5.47)
*R*_merge_ (%)	7.2 (93.3)	17.2 (97.5)
<*I*/σ(*I)*>	17.88 (2.05)	5.25 (1.16)
Refinement		
*R*_free_ reflections	1043	1872
No. of atoms (non-H)		
protein	7276	7063
ligands	139	175
solvent	1278	1190
*R*_work_/*R*_free_ (%)	11.9/15.6	18.4/21.9
Mean ADP^a^ (Å^2^)	19.8	20.6
RMSD from ideal geometry		
bond lengths (Å)	0.012	0.011
bond angles (°)	1.7	1.8
Ramachandran statistics (%)		
favored	96	97
allowed	3	3
outliers	1	0
PDB code	8QEU	8QEV

a ADP, atomic displacement parameter.

Values in parentheses refer to the highest-resolution shell.

### Structure determination and refinement

Initial structure of unliganded *At*OTC was solved with Phaser (McCoy et al., 2007) using *Pyroccocus furiosus* OTC (PDB ID:1PVV) as a model (Massant et al., 2003). The Phaser solution was rebuilt using AutoBuild (Terwilliger et al., 2008) from the PHENIX (Liebschner et al., 2019) package. Afterwards that structure underwent both manual and automatic refinement interchangeably. Manual refinement was carried out using Coot (Emsley et al., 2010) and automatic refinement using Refmac5 (Murshudov et al., 2011) of CCP4 package (Agirre et al., 2023). The structures of complexes were solved using Phaser with unliganded structure of *At*OTC as a model and underwent the same refinement protocol. At later stages of refinement, the complex of *At*OTC with ornithine was refined using anisotropic B-factors and complex with carbamoyl phosphate was refined using TLS parameters (Painter and Merritt, 2006a) generated by TLSMD server (Painter and Merritt, 2006b). Quality of refinement was controlled by R_work_ and R_free_ (Kleywegt and Jones, 1997). Refinement statistics are provided in **Table 1**.

*At*OTC naturally consists of 375 amino acids (UniProt ID: O50039). Since it acts in the chloroplast, the construct was devoid of the first 53 residues, based on the analysis with TargetP (Almagro Armenteros et al., 2019). Furthermore, tracing of additional 19 residues was not possible due to lack of electron density in the map, hence the structure begins at L72. In the complex structure with ornithine all further residues have been modeled into the electron density map, whereas in carbamoyl phosphate bound structure the SMG loop is unstructured and lacks electron density map. There are three Ramachandran outliers in each chain, P176, L196 and V340, all of which fit perfectly into the electron density map.

The molecular representation of presented structures was done using UCSF Chimera (Pettersen et al., 2004); the secondary structure elements were recognized with PDBSUM1 standalone version (Laskowski, 2022).

### Phylogenetic analysis of aspartate/ornithine carbamoyltransferase superfamily sequences

A set of 1080 *Viridiplantae* (taxid: 33090) sequences annotated and assigned to aspartate/ornithine carbamoyltransferase superfamily (IPR036901) were downloaded from InterPro database (Finn et al., 2017) for the phylogenetic analysis. Duplicated sequences have been removed using ElimDupes (https://www.hiv.lanl.gov/content/sequence/elimdupesv2/elimdupes.html). Ambiguous sequences containing non-canonical characters, such as X, have been deleted. In order to remove clear outliers (incomplete and wrongly annotated sequences) the mean length of the sequences and its standard deviation were calculated. On this basis, records with the length of mean ± SD were subjected to further analysis. This set contained 822 unique sequences that were aligned in MAFFT (Katoh and Standley, 2013) and further analyzed with EFI - ENZYME SIMILARITY TOOL (Zallot et al., 2019) to create a sequence similarity network in order to group related proteins into clusters. The final network with 325 575 edges was prepared with the alignment score of 124 and E-value for edge calculation of 5. The network was analyzed with Cytoscape (Shannon et al., 2003). Sequences were grouped into two clusters containing 389 OTCs, 377 ATCs, and 56 sequences not assigned to any cluster. Since most of the 56 outliers lack characteristic features of carbamoyltransferases, they were excluded from further analysis. 389 OTC sequences were then used for the inspection of sequence conservation in ConSurf (Yariv et al., 2023).

For proteins which have their structure deposited in the PDB, sequence analysis was prepared. A list of structures of proteins belonging to aspartate/ornithine carbamoyltransferase superfamily was downloaded from InterPro database (179 entries). It was used to download full protein sequences from UniProt (244 entries) (Consortium, 2022). Then, sequences with an identity greater than 99% were removed using ElimDupes (www.hiv.lanl.gov/content/sequence/elimdupesv2/elimdupes.html). This procedure filtered engineered mutants. Sequences of fusion CAD proteins spanning over 2000 residues as well as regulatory proteins, not exhibiting enzymatic activity have been excluded from the analysis (UniProt ID: P27708, G0S583, P0A7F3, P74766, D0VWV9, O66990). Sequences of *Ps*OTC, *Ps*ROTC and *At*OTC were added even though no structures of theirs were published. Sequence alignment of the remaining 41 sequences was done using ClustalW (Thompson et al., 1994) in MEGAX (Kumar et al., 2018). The phylogenetic tree was prepared using TreeViewer (Bianchini and Sánchez-Baracaldo, 2023) with UPGMA algorithm.

## Data availability statement

The datasets presented in this study can be found in online repositories. The names of the repository/repositories and accession number(s) can be found below: https://www.rcsb.org/, 8QEU https://www.rcsb.org/, 8QEV.

## Author contributions

MN: Conceptualization, Data curation, Formal analysis, Investigation, Visualization, Writing – original draft, Writing – review & editing. AP-B: Data curation, Formal analysis, Writing – original draft, Writing – review & editing. AW: Data curation, Formal analysis, Writing – review & editing. BS: Conceptualization, Data curation, Formal analysis, Investigation, Supervision, Validation, Writing – original draft, Writing – review & editing.
