# Supplementary material for: Structural analysis and molecular substrate recognition properties of Arabidopsis thaliana ornithine transcarbamylase, the molecular target of phaseolotoxin produced by Pseudomonas syringae
Source: Front Plant Sci. 2023 Dec 18;14:1297956. doi: 10.3389/fpls.2023.1297956 (PMC10765591; doi:10.3389/fpls.2023.1297956)
Supplement: Supplementary file 1 [file Image_1.pdf]

SUPPLEMENTARY INFORMATION

Structural analysis and molecular substrate recognition properties of *Arabidopsis thaliana* ornithine transcarbamylase, the molecular target of phaseolotoxin produced by *Pseudomonas syringae*

Maciej Nielipinski[1], Agnieszka J. Pietrzyk-Brzezinska[1], Alexander Wlodawer[2], Bartosz Sekula[1]*

[1]Institute of Molecular and Industrial Biotechnology, Faculty of Biotechnology and Food Sciences, Lodz University of Technology, Lodz, Poland

[2]Center for Structural Biology, National Cancer Institute, Frederick, MD, United States

***Correspondence:**

Bartosz Sekula

Institute of Molecular and Industrial Biotechnology

Lodz University of Technology

Stefanowskiego 2/22

90-537 Lodz, Poland

+48 42-631-34-41

bartosz.sekula@p.lodz.pl

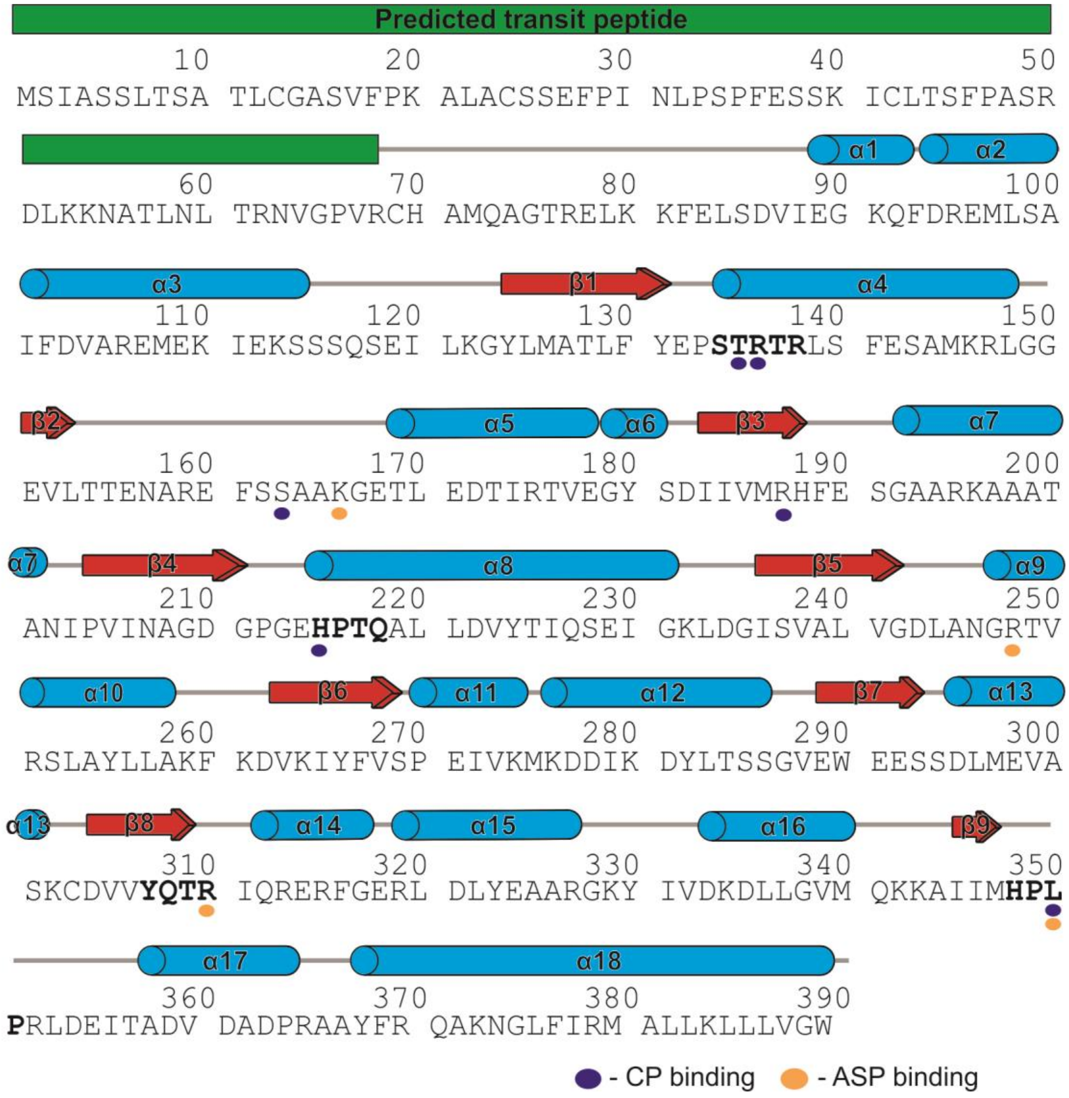

Supplementary Figure S1. ***Arabidopsis thaliana*** **aspartate transcarbamylase (*At*ATC)**. Secondary structure and binding sites of *At*ATC mapped on the primary structure of *At*ATC (UniProt ID: P49077). Conserved motifs are bold. Secondary structure elements are shown as blue pipes (helices); red arrows (β-strands), and grey lines (loop regions); the green solid cuboid depicts the predicted transit peptide. Residues interacting with CP and ASP in the active site are marked with purple and orange dots.
